# Harnessing Maillard reaction byproducts for dual emissive carbon quantum dots: a tunable optical platform

**DOI:** 10.1039/d5ra04569j

**Published:** 2025-08-28

**Authors:** Farwa Nurjis, Rafaqat Ali, Hina Ali

**Affiliations:** a National Institute of Lasers and Optronics College, Pakistan Institute of Engineering and Applied Sciences Nilore 45650 Islamabad Pakistan hinali991@hotmail.com

## Abstract

Quantum dots (QDs) have diverse applications, ranging from optics and energy to biomedical. In this study, carbon quantum dots (CQDs) were synthesized using glucose and tryptophan as precursors using one-step microwave (MW) and sand bath (SB) thermal methods, and the CQDs exhibit distinct photoluminescence behaviors. CQD-SB shows enhanced and stable fluorescence despite its amorphous structure, likely due to prolonged thermal treatment, facilitating the formation of robust surface states and stable reaction products. Notably, CQD-SB generates a dual emissive bands activated at both shorter and longer excitation wavelengths (330–390 nm) reveals both core-localized and surface bound group emission. This stable dual emission suggests a hybrid fluorescence mechanism involving excitation, concentration and size-dependent effects. However, CQD-MW possesses a partially crystalline structure and exhibits excitation-dependent dual emission even at higher excitation energies, showing less stability. This behavior of CQD-MW is due to rapid carbonization and limited passivation owing to instant microwave heating. Fluorescence staining reveals that CQD-SB offers stronger and more stable blue and green emission in human buccal and onion epidermal cells, supporting its potential as an efficient bioimaging probe and alternative to synthetic dyes.

## Abstract 2

Quantum dots (QDs) have diverse applications, ranging from optics and energy to biomedical.

## Graphical abstract

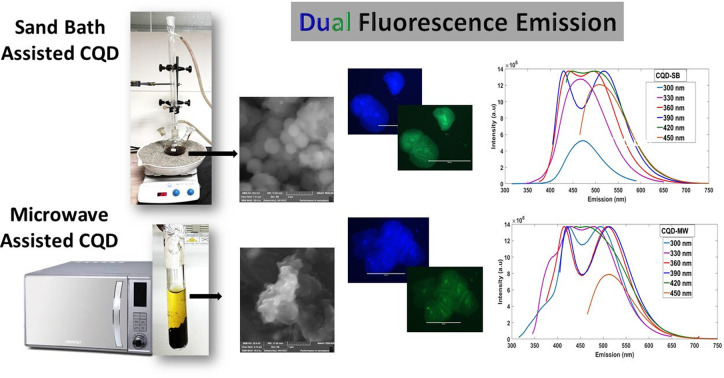

## Introduction

Advances in nanoscience and technology have opened new applications for nanomaterials, such as fluorescent carbon dots (CDs),^1^ due to their unique properties such as solubility, photoluminescence, biocompatibility, photo-stability, and fluorescence emission. These materials are promising for optical devices, bio-imaging, catalysis, drug delivery, sensing, and medical diagnosis.^2–5^ CDs, reported for the first time in 2004, based on their consistency, diversity of the carbon core and inherent chemical, physical, and optical properties, can be divided into carbon nanodots (CNDs), graphene quantum dots (GQDs), graphitic carbon nitride quantum dots (g-CNQDs), carbon quantum dots (CQDs), and carbonized polymer dots (CPDs).^6^ As a type of zero-dimensional, spherical nanomaterial, carbon quantum dots are tiny carbon nanoparticles (NPs) that are less than 10 nm in size,^7^ exhibiting an amorphous core shell structure with mixed sp^2^/sp^3^ hybridization or a graphitic crystalline structure with sp^2^ carbon.^8^ They possess hydroxyl (–OH), carboxyl (–COOH), aldehyde (–CHO), amino (–NH_2_) and sulfhydryl (–SH) groups on their surface.^1^ CQDs formation mechanisms rely on the synthesis strategy, precursors and reaction conditions.^9^

CQDs can be synthesized from carbohydrates, proteins, lipids, and other biological molecules, as well as from renewable sources, such as fruit and vegetable peels, nuts, and waste materials, containing –OH, –COOH, and –NH_2_ functional groups using a bottom-up approach, including hydrothermal/solvothermal^10,11^ combustion, pyrolysis,^12^ and microwave irradiation^13^ techniques. Alternatively, using top-down methods, CQDs can be synthesized from pure carbon of different forms using different techniques, such as laser ablation,^14^ electrochemical^15^ and arch discharge methods.^16^ Researchers extensively favor using bottom-up approaches due to their simple synthesis, controllable reaction conditions, and inexpensive raw materials, allowing for one-step high-volume synthesis of CQDs with high QY and excellent optical properties.^17^ CDs that exhibit dual emission upon single-wavelength excitation cannot be obtained using a top-down technique; dual emission is highly advantageous in CQDs multipurpose applications.^18^

Microwave-mediated syntheses are one of the simple bottom-up approaches used to synthesize CQDs. Microwaves are electromagnetic waves that have a frequency between 0.3 and 300 GHz. Heterogeneous temperature distributions within a sample are commonly observed in microwave-mediated synthesis, which relies on the transfer of heat by convection or conduction.

From the hotter outer sample to the cooler center, the ensuing temperature differential may cause incomplete reactions or encourage the production of byproducts from competitive side reactions.^19^ Because of its straightforward design and dependable performance, the sand bath method is a useful and efficient thermal technique for synthesizing CQDs. Another less utilized bottom-up process is the sand bath, which provides uniform heating without direct flame or microwave exposure, making it ideal for the thermal decomposition or carbonization of chemical precursors. Sand bath is a simple, adaptable process that allows longer reaction times and minimizes localized overheating and better thermal stability, making it valuable for green and chemical-based CQDs synthesis in research and teaching laboratories.^20^

Pure carbon or mostly carbon, oxygen, and hydrogen-based multi-emissive CQDs showed low fluorescence and quantum yields (QYs).^21^ This restriction can be addressed by improving their fluorescence by changing their electrical and photophysical characteristics, increasing their functional adaptability, and expanding their range of uses, most frequently by N-doping with amino acids.^6^ As nitrogen serves as an efficient dopant because of its atomic size, which closely resembles that of carbon, its strong electronegativity, the five valence electrons available for chemical bonding, and a lone electron pair can be easily transported to the orbitals of sp^2^ carbon structures.^22^ Nitrogen-doped carbons have been synthesized using various methods, including the post-treatment of carbon with amines (or urea), ammonia, as well as more direct methods utilizing acetonitrile, pyrrole, polyacetonitrile, or polyaniline as starting materials or amino acid precursors.^23^ When reducing sugars and amino groups are heated at high temperatures, a non-enzymatic browning reaction takes place. The proposed mechanism for the formation of CQDs was based on the Maillard reaction, which is the reaction between sugars and amino group-containing molecules to produce brown polymeric or heterocyclic nitrogen compounds known as ‘melanoidins’ after a series of complex reactions, including dehydration, rearrangement, isomerization and condensation.^24^ Reactions involving dehydration, decarboxylation, deamination, and/or dehydrogenation combine to form the carbonization process. The precursor's molecular structures and reaction temperature are the main determinants of these reactions. As the degree of carbonization increases, the C content also increases. The sp^2^ domains in the carbon core grow as a result of increased carbonization. Carbon cores and surface groups make up the majority of fully carbonized CDs.^18^

The size of π-conjugated domains, the types and contents of surface groups, and variations in the oxygen and nitrogen content of carbon cores affect the absorption characteristics of CQDs.^25^ Furthermore, the relationships between the excitation-dependent/independent emission feature and the complicated structures of CQDs depend on the particle sizes, sp^2^ carbon framework, graphitization degree, heteroatom doping, organic groups and surface states.^26^ The optical properties of CQDs can be tuned by varying the carbon sources and reaction conditions, as the photoluminescence (PL) features of CQDs correlate with their particle sizes, element contents, and functional groups.^27^ The optical performances of CQDs are determined by both their carbon cores and surface states, so optimizing the dehydration reaction between precursors and adjusting the graphitization degree produces CQDs with desired PL properties.^28^

Multicolor emission, two-/multiphoton photoluminescence, high photostability, excellent biocompatibility, simple synthetic routes, and flexible designability make CQDs next-generation fluorescent probes for both *in vitro* and *in vivo* bioimaging as reported for imaging cells, microorganisms,^29^ and plant tissue.^30^ Generally, CQDs can quickly enter cells through energy-/temperature-dependent macropinocytosis, clathrin, caveolae, and lipid raft-mediated endocytosis, resulting in their distribution in mitochondria, lysosomes, endoplasmic reticulum, Golgi apparatus, and/or nucleolus,^31–34^ based on the different nanostructures of CQDs and types of cells. Currently, physical mixing,^35^ nanohybrid,^36^ and dual-emission CQDs^37^ are the three primary methods for constructing CQD-based ratiometric fluorescent probes. The direct dual emission from CQDs without the further addition of luminous elements may be due to the result of their intrinsic state emission and surface energy traps,^38^ or the co-doped atom may cause dual emission by creating an additional orbital energy gap that allows valence electrons to move freely.^39^ The optical characteristics of CQDs can be influenced by their interparticle distance in addition to their initial emission; a long CQD interparticle distance results in blue-emissive states, while a close CQD distance promotes green emission.^37^

This study aims to synthesize CQDs using simple precursors, such as glucose and tryptophan along with the comparison of two synthesis procedures, *i.e.* using house hold microwave and sand bath. The physicochemical characteristics of the produced CQDs were systematically investigated, including their morphological features, composition, structure, fluorescence and photoluminescence behavior up to the single cell stage.

## Experimental section

### Materials and methods

d-Glucose, tryptophan and ethanol of reagent grade were purchased from Sigma Aldrich (St. Louis, MO, USA). In every experiment, aqueous solution preparations were made using ultra-pure deionised water (Milli Q). The fluorescence of the samples was observed under a UVA lamp (315–400 Uvac, ICCC, Pakistan). CQDs were characterized by ultraviolet-visible (UV-vis 2200, Rayleigh, China), fluorescence (Horiba Flouromax 4, USA) and Fourier-transform infrared (FTIR) (Bruker ATR-FTIR, U.S.A) spectroscopy. The shape and morphology were studied by Scanning Electron Microscopy (SEM), (TESCAN, MAIA 3, Czech Republic). Bioimaging was performed using fluorescent microscopy (Evos® FL Cell Imaging System; Thermo Fisher Scientific, MA, USA).

## Experimental design

### Synthesis of carbon quantum dots

In this study, two commonly used bottom-up approaches, house hold microwave and setting up the reflux setup on a sand bath, were compared to synthesize the CQDs using simple precursors, *i.e.* glucose and tryptophan.

#### Sand bath synthesis of CQDs

16 g of d-glucose and 2 g of tryptophan were dissolved in 50 mL of deionized water in a round bottom flask. The round bottom flask was adjusted under the reflux apparatus and placed on a sand bath at 250 °C under magnetic stirring. The reaction was continued for 24 h until the solution gradually turned brown. The supernatant of the resulting CQD-SB solution was collected and stored at 4 °C for further analysis.^40^

#### Microwave-assisted synthesis of CQDs

d-Glucose, tryptophan and water with the above-mentioned ratio were subjected to heating using a house hold microwave oven for 45 min until complete browning. The oven has an output power of 900 W with a 2450 MHz frequency and temperature around 273 °C. The water was thoroughly mixed in the dried carbonized material after cooling to room temperature, and the supernatant was kept at 4 °C for further analysis.^41^

## Characterization

### Morphological analysis

#### Scanning electron microscopy

The morphology was analyzed under SEM (TESCAN, MAIA 3, Czech Republic). The lyophilized samples were placed on carbon tape, and an accelerating voltage of 15 kilovolts was used to capture the micrographs of each sample, which carry information about the structure and topography of the sample.

#### Dynamic light scattering

Dynamic light scattering (DLS) was used to examine the hydrodynamic diameter, polydispersity index (PDI) value, and zeta potential of CQD-SB and CQD-MW. The samples were diluted (1 : 1000) with water and analyzed using a DLS analyzer (Microtrac Nanotrac Wave II, USA). The analysis was carried out in triplicate at 25 °C, and the data were analyzed using Microtrac Flex 11.1.0.1 software.

### Elemental analysis

#### Fourier transform infrared spectroscopy

The functional groups of CQD-SB and CQD-MW were studied using an FTIR spectrophotometer (Thermo Nicolet, USA) equipped with OMNIC version 6.0 software. The scanning speed was maintained at 16 scans per spectrum, and the measurement range was adjusted between 400 and 4000 cm^−1^.^42^

#### X-ray diffraction spectroscopy

Both freeze-dried CQDs were subjected to X-ray diffraction (XRD) studies^43^ to analyze their crystallographic properties and physical state in a range of Bragg angles *θ* (5–90°) at a scanning rate of 2*θ* min^−1^ (Bruker AXS, Inc.).

### Optical properties

#### Paper chromatography

The drops of supernatant and the sediments of both CQD-SB and CQD-MW were placed on cellulose filter paper and air dried. Furthermore, the supernatant of CQD-SB and CQD-MW was passed through 0.22 μm syringe filters, and the drops from these filtrates were also placed on the filter paper. Additionally, the drops from these diluted filtrates (2.5 μg mL^−1^) of both CQDs were placed on cellulose filter paper to observe the migrated CQDs. The fluorescent zones of CQDs were observed after drying these filter papers and imaged under UVA (315–400 nm) using a 2UV-transilluminator MERADD (ICCC).

#### Ultraviolet visible (UV-vis) spectroscopy

The UV-vis spectra were recorded to examine the optical properties of the synthesized CQD-SB and CQD-MW in the range of 200–600 nm (UV-vis 2200, Rayleigh, China). The CQDS were placed in a quartz cuvette with a 10 mm optical path to obtain the absorption spectra at a scan rate of 1.0 nm. This spectroscopic method works on the basic principle that chemical substances interact with visible or ultraviolet light. Different and distinctive spectra are produced as a result of the excitation and subsequent de-excitation processes of materials during light absorption.

#### Photoluminescence studies

CQD-SB and CQD-MW were subjected to photoluminescence (PL) using the conventional emission with excitations at 300, 330, 360, 390, 420 and 450 nm. In addition to excitation-dependent PL emission, concentration-dependent PL at 330 nm was also conducted for both CQDs using concentrations ranging from 0.1 to 40 mg mL^−1^. PL data were acquired using the Fluoromax-4 fluorescence spectrophotometer with excitation and emission slit sizes of 5 nm. Synchronous fluorescence spectra (SFS) were also acquired for both CQDs by scanning with a constant wavelength difference (Δ*λ*) of 30 nm in the range of 200–600 nm. SFS narrows spectral bands by scanning excitation and emission wavelengths simultaneously while keeping a fixed gap (Δ*λ*) between them, resulting in improved spectral clarity. This approach simplifies complex fluorescence spectra and aids in the identification of specific fluorophores or components within intricate samples.

#### Quantum yield estimation

To calculate the quantum yield (QY) of the CQD-SB and CQ-MW, two reference fluorescent dyes were used, which fluoresce in the blue and green regions. The emission spectra of the ethanolic solution of Coumarin 450 and Fluorescein were obtained when excited at 350 nm and 460 nm, respectively. The UV-vis absorbance values of both CQDs were compared to those of Coumarin 450 (0.94 or 94% quantum yield) and Fluorescein (0.97 or 97% quantum yield). The integrated fluorescence intensity is defined as the area under the PL curve spanning the wavelength range of 365–690 nm and 475–910 nm for C-450 and Fluorescein, respectively. A plot of the integrated fluorescence intensity against the absorbance value was created for both C-450 and Fluorescein.

The quantum yield was obtained using the following equation:(QY)_S_ = (QY)_R_ (PL_area_÷ OD)_S_/(PL_area_÷ OD)_R_ × *η*^2^S/*η*^2^R,where QY_S_ is the quantum yield of CQD-SB and CQD-MW, QY_R_ is the quantum yield of Coumarin 450 and Fluorescein OD is the optical density, PL_area_ is the area under the fluorescence emission peak, and *η* is the refractive index of the ethanol solvent (1.361). The subscripts “R” and “S” stand for reference substance and sample, respectively. The absorbance in a 10 mm quartz cuvette is maintained below 0.1 OD to reduce the effects of re-absorption when excited at 350 and 460 nm.

#### Machine learning

Simple gradient descent and conjugate gradient approaches are outperformed by applying the popular Levenberg–Marquardt (LM) algorithm. However, it is locally convergent and iteratively diverges when the initial guess is poor. The GA results in a parameter estimation value, *X k*, for the LM algorithm's preliminary solution, which is applied to the training, validation, and test phases of quantum dot fluorescence intensity disintegration modeling and nonlinear decay kinetics.

### Single cell imaging studies

#### Fluorescence imaging of a plant cell

The small piece of onion epidermal membrane was separated carefully and placed on the glass slides, and 2 drops of 2.5 μg mL^−1^ solutions of CQD-SB and CQD-MW were placed on it, following the protocol described^32^ with certain modifications. The slides were washed with absolute ethanol after an hour and covered with a thin glass coverslip, ensuring that there was no air bubble and imaged under a cell imager (Evos® FL Cell Imaging System; Thermo Fisher Scientific, MA, USA) using 3 modes of transmittance *i.e.* white light, 357 nm and 395 nm excitation.

#### Fluorescence imaging of buccal mucosa

A sample of buccal mucosa was microscopically observed under a cell imager (Evos® FL Cell Imaging System; Thermo Fisher Scientific, MA, USA) after being incubated with a 2.5 μg mL^−1^ concentration of CQD-SB and CQD-MW. The samples were washed with absolute ethanol and subjected to imaging on a glass slide after an hour, following the protocol with certain modifications, as described.^44^

## Results and discussion

### Synthesis of CQD-SB and CQD-MW

In this study, two facile and cost-effective bottom-up synthesis approaches, *i.e.* the house hold microwave irradiation and conventional sand bath heating techniques, were compared to synthesize the excitation-dependent dual emissive carbon quantum dots (CQD-SB and CQD-MW) using simple precursors, such as glucose and tryptophan. The aqueous solution of both CQDs showed a combination of blue and green fluorescence when illuminated under UVA (315–400 nm). The synthesized CQD-SB showed a dark brown color with noticeable thick sediment at the bottom of the container. Similarly, in CQD-MW, the Glu–Trp solution evaporated completely, resulting in phase separation upon redispersion; clear pale-colored supernatant and dark brown sediment were observed.

Precursors typically contain polymerizable moieties in their skeleton that can take part in condensation/addition polymerization processes, such as hydroxyl groups (–OH), carboxyl groups (–COOH), amino groups (–NH_2_), and carbon double bonds (–C=C).^10^ When an aqueous solution of glucose and tryptophan was heated at 250 °C for 24 h in the case of a sand bath and 273 °C (900 W) in a microwave for 45 minutes, the carbonyl group on glucose and the amino group on tryptophan condense to produce N-substituted glycosylamine. Amadori rearrangement of the glycosylamine molecule yields ketosamine, which is subsequently dehydrated and fragmented to yield N-free chemicals, including furfurals, aldehydes, aldols, and N-free polymers. Finally, heterocyclic nitrogen molecules, including pyridines, pyrazines, and pyrroles, are produced by aldehyde-amine condensation. These compounds then undergo carbonization to create CQDs with nitrogen-incorporated structures. Both precursors were selected carefully in the optimal ratio using a longer reaction time so that the dehydration reaction under supercritical conditions favored the formation of conjugated sp^2^ domains and dope nitrogen into the domains.^45^ At the initial reaction stage, the newly formed crosslinking molecules are amorphous and unclear and then agglomerate and become darker in color; finally, carbonized nanoparticles are formed. This formation process indicates that the dehydration reactions among the precursors first produced Maillard Reaction Products (MRPs) and then turned into carbon quantum dots. The particle size and graphitization degree of CQDs highly depend on dehydration and carbonization conditions.^32^ The microwave radiations produce heating using two mechanisms owing to ionic conduction and dipolar polarization. Ionic conduction, influenced by charged particles, affects the dipolar polarization effect of reaction mixture dipoles. When an electric field is applied, ions align with the field, causing dielectric loss and molecular friction.^46^ In contrast, the sand bath technique is better than the microwave technique owing to the uniform provision of heat; moreover, it neither involves any strong acid or base for the initiation of carbonization nor any surface passivating agent.^47^ The condensation reaction, which takes place during the advanced stage of the Maillard reaction, closely resembles the processes that occur during reflux-based sand bath synthesis of CQDs made from organic precursors.^40^

### Morphological properties of SEM and DLS analyses

**Fig. 1:**
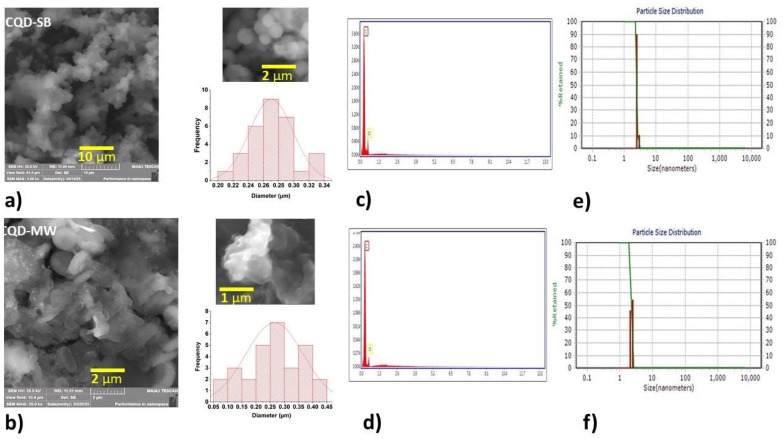
(a) and (b) SEM images of micro-sized colloidal CDs in CQD-SB and CQD-MW, along with the histograms representing the frequency of the observed particle diameters in μm, overlaid with fitted Gaussian curves to illustrate distribution symmetry and spread; (c) and (d) EDX pattern of CQDs showing C and O as main elements; (e) and (f) DLS data showing particle size distribution.

For as-prepared CQD-SB and CQD-MW, when examined under SEM after freeze drying, both CQDs appear colloidal in nature with spherical structures of different sizes (Fig. 1a and b). The histograms generated from the SEM images represent the size distribution profiles of both CQDs, as shown adjacent to the SEM images (Fig. 1a and b). The CQD-SB demonstrates a narrow and more symmetric distribution, ranging from 0.22 to 0.34 μm, with a pronounced peak at around 0.27 μm, which indicates a more uniform particle population. In contrast, the CQD-MW sample exhibits a broader size distribution, spanning from approximately 0.05 to 0.45 μm, with the modal size of around 0.25–0.30 μm. The wide distribution suggests a higher degree of size heterogeneity, and micron-sized particles reflect potential aggregation during the freeze drying process. The negative surface charge for CQD-SB was probably due to carboxyl-rich surface groups, while CQD-MW displayed a positive charge, suggesting the presence of amine or amide groups. These disparities in surface charge and size dispersion may influence the colloidal stability, fluorescence properties and potential applications of the CQDs.

Nanoparticles tend to agglomerate and form clusters at high concentrations. The nanoparticle aggregation resulted in a gradual increase in particle size, which increased the electron-conjugated π-domain structure, thereby generating a new radiation recombination path, which resulted in a change in fluorescence generated from CQD-SB and CQD-MW.^48^ The EDX data also confirmed the presence of carbon and oxygen as the main elements in both CQDs (Fig. 1c and d).

DLS analysis revealed that both CQD-SB and CQD-MW are well-dispersed in aqueous media. Diluted samples (1 : 1000) of both CQDs for dynamic light scattering studies exhibited CQD-SB with a mean diameter of 2.338 nm, with 0.0549 PDI and 14.6 mV zeta potential with negative polarity. CQD-MW depicted 2.518 nm, with 0.012 PDI and 21.8 mV zeta potential with positive polarity (Fig. 1e and f). A low value of polydispersity indexes indicates a monodisperse population; however, zeta potential measurements showed a negative surface charge for CQD-SB probably owing to carboxyl-rich surface groups, while CQD-MW displayed a positive charge, suggesting the presence of amine or amide groups. These differences in surface charge and dispersion behavior may influence the colloidal stability, fluorescence properties and potential applications of the CQDs. The change in size and morphology may be due to the degree of carbonization as observed when l-serine and l-tryptophan were heated at 100 °C and formed annular dots; at 200 °C, spherical particles with an amorphous carbon core were formed, and at 300 °C, they became the graphitic carbogenic particles.^49^

The particle size difference related to SEM and DLS was due to the use of lyophilized particles while performing SEM, while DLS measurements were conducted in water used as a liquid medium. The sizes obtained from SEM depict the distinct morphology of the CQDs, while DLS reflects the dynamic size distributions that can vary with sample dilution and stability.

SEM revealed distinct differences in size distribution between CQD-SB and CQD-MW, with CQD-SB, showing more uniform particle formation due to gradual sand bath heating. In contrast, CQD-MW showed a broader and less symmetrical distribution, reflecting heterogeneous nucleation and growth dynamics likely due to instant microwave heating. The photophysical behavior and stability of the CQDs are influenced by these differences in size uniformity and dispersion, as discussed in the subsequent sections. We also noticed that an increase in CQD concentration leads to increased aggregation, causing larger diameters in the DLS measurement. This concentration-dependent clustering also aligns with changes in fluorescence emission patterns on paper chromatography, as discussed below in Fig. 3i–l, indicating inter-particle interactions. Even though the absolute size measurements differ between the two techniques, they together provide a complementary perspective on particle behavior in both dry and solution states and confirm their concentration-dependent nature.

### Elemental properties

#### FTIR analysis

**Fig. 2:**
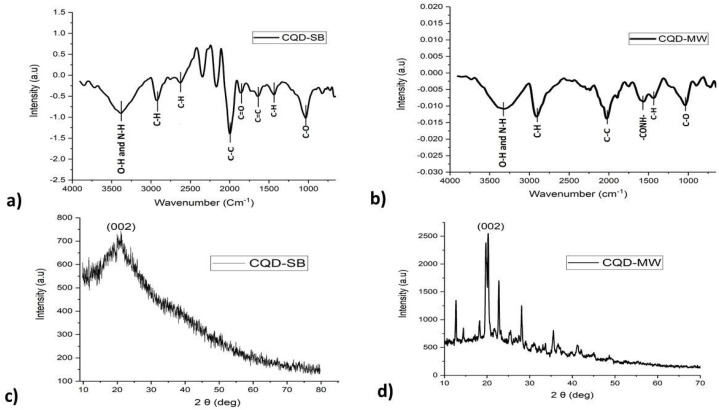
(a) and (b) FTIR absorption spectra of CQD-SB and CQD-MW; (c) and (d) XRD pattern shown by both CQD-SB and CQD-MW as amorphous powder.

The FTIR spectrum showed the presence of a strong band at 3075–3420 cm^−1^ assigned to O–H/N–H stretching vibration of hydroxyl and amine groups, which can be clearly observed in both CQD-SB and CQD-MW (Fig. 2a and b). However, CH stretching vibration bands were observed at 2600–2956 cm^−1^ in CQD-SB, while CQD-MW showed a single band. CQD-SB displayed a strong C–C peak at 1993 cm^−1^, while low intensity peaks at 2026 cm^−1^ were observed in CQD-MW. The presence of C=C and C–H is indicated by the peaks in CQD-SB at 1649 cm^−1^ and 1435 cm^−1^, respectively. The presence of aromatic cyclic C=C stretching vibration is responsible for the peak at 1636 cm^−1^.^47^ The peak of –CONH– stretching vibration was observed at 3378 cm^−1^ and 3320 cm^−1^ for CQD-SB and CQD-MW, respectively. The stretching vibration of the C–H bond is observed at 2936 cm^− 1^ and 2624 cm^−1^ for CQD-SB and 2913 cm^−1^ for CQD-MW, respectively. Peaks observed at 1031 cm^−1^ and 1045 cm^−1^ for CQD-SB and CQD-MW, respectively, because of the C–O bond, while the peak at 1857 cm^−1^ for C=O carbonyl stretching vibrations can be observed only in CQD-SB.

The infrared spectra of CQD-SB and CQD-MW showed slight variations with a substantial number of hydroxyl, carboxyl, amino, or amide groups, indicating the formation of CQDs with carbon cores and polyaromatic domains conferring hydrophilicity, good dispersibility and high stability in an aqueous solution.

#### XRD analysis

The crystallinity and degree of graphitization of the CQD-SB and CQD-MW were assessed using XRD. The X-ray diffraction (XRD) pattern of CQD-SB revealed a broad (002) reflection, showing a disordered stacking and amorphous structure, and a sharp peak centered at 2*θ* = 20.4° with an interlayer spacing of 0.444 nm for CQD-MW, indicating a partial crystalline structure (Fig. 2c and d). The results suggest that the amorphous structure of CQD-SB is likely due to the presence of oxygen and a hydrogen/nitrogen functional group bonded on the edge of the basal plane. In our case, the lattice spacing of carbon dots is higher than the graphitic interlayer spacing (0.33 nm) close to the graphite (002) plane, indicating the presence of oxygen-containing groups with sp^3^ carbon and hydroxyl-functionalized carbon domains integrated in the hexagonal graphite lattice studied previously.^45^

However, CQD-MW shows a relatively sharper and intense peak (002) at the same angle, suggesting partial crystallinity and improved graphitic domain formation likely attributed to rapid heating in microwave-assisted synthesis. Both sand bath-heated and microwave-heated CQD samples undergo Maillard reactions during thermal treatment; however, prolonged heating in CQD-SB favors extensive condensation and carbonization, leading to sheet-like, amorphous structures. The intense peaks in the PL spectra of CQD-SB (discussed below) challenge the general assumption that amorphous CQDs are less emissive, highlighting the dominant role of surface chemistry over core crystallinity in photoluminescence behavior.^50^ Moreover, microwave-assisted synthesis induces rapid nucleation and localized heating, forming toroidal (donut-like) carbon clusters, as mentioned in the SEM image (Fig. 1b). The higher content of O and N containing groups in CQD-SB causes stearic hindrances and forms sp^3^ bonds that disrupt the sp^2^ domain, thus causing structural disorders. Therefore, the precursor, the addition of dopant and the reaction time also played a role in such a difference, as depicted in XRD patterns. These morphological variations reflect the distinct thermal kinetics and dopant incorporation profiles of each method.

#### Optical properties

The electronic structures and optical properties of the CQD-SB and CQD-MW were investigated using paper chromatography illumination, UV-vis absorption spectroscopy, synchronous florescence and PL excitation and emission spectroscopy.

**Fig. 3:**
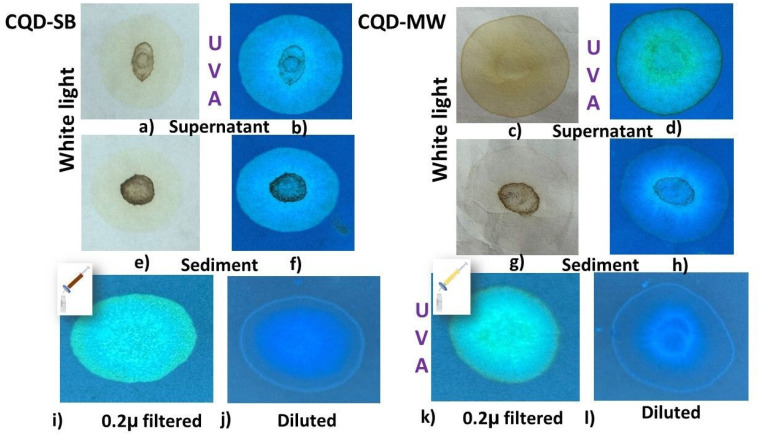
(a)–(d) Paper chromatography of CQD-SB on the left side and CQD-MW on the right, showing drops placed on filter paper in white light and UVA. (e)–(h) Drops from CQD sediment on filter paper. (i)–(l) Both CQDS were filtered (0.2 micron); the filtrate was diluted with water, and drops placed on filter paper fluoresced under UVA, showing a typical difference in color of the filtrate; and diluted filtrate showed concentration-dependent fluorescence of CQDs.

The CQD's supernatant, sediment, filtrate (200 nm) and dilutions were separated using a chromatographic technique with cellulose filter paper and illuminated under UVA. Interestingly, the drops from the aqueous solution (2.5 μg mL^−1^) of both CQD-SB and CQD-MW placed on the filter paper and exposed to UVA light produced a stunning gradient of cyan, blue and blueish green colored patterns owing to the capillary action (Fig. 3). Fig. 3 illustrates the white light and UVA illuminated images of the supernatants and sediments of CQD-SB and CQD-MW, respectively. The final MRPs did not show any intrinsic fluorescence, but blue emission originated from the supernatant and particles found in the sediment of CQD-SB (Fig. 3b and f). However, emission with a greenish hue originated from the center of the CQD-MW supernatant, while the sediment fluoresces bright blue (Fig. 3d and h). This size-dependent fluorescence, as explained by the paper chromatography of the CQD-SB of the filtrate, resulted in cyan fluorescence under UVA, followed by blue emission upon dilution, strongly suggesting quantum confinement. Smaller CQDs typically emit at shorter wavelengths (blue), while larger ones shift toward green/yellow due to a reduced bandgap. In contrast, the blue-centered emission in CQD-MW was also observed upon the dilution of the filtrate (Fig. 3k and l), implying size-dependent quantum confinement, consistent with core-derived fluorescence. The concurrent cyan ring formation along with blue fluorescence suggests that the attribution of incomplete passivation represents the surface bound emissive states.

**Fig. 4:**
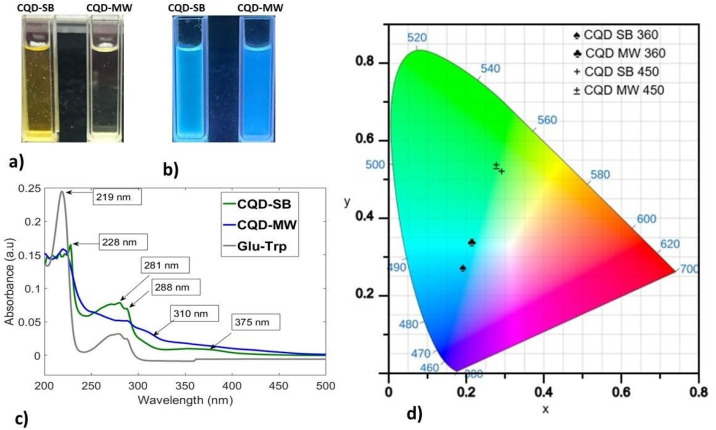
(a) and (b) CQDs produced through sand bath (CQD-SB) and microwaves (CQD-MW) under white light and fluorescent blue under UVA. (c) Absorption spectra of both CQDs and precursors (glucose and tryptophan). (d) Variation coordinates in the blue and green regions in the CIE chromaticity diagram of both CQDs with excitations at 360 and 450 nm.

The pattern of color separation in both CQDs corresponds to the particle size. Such optical properties of diluted and concentrated carbon dots concur with a previous study,^51^ which relates to the distinct chemical and visual characteristics of large aggregates in the reaction mixture, reflecting the transformation of chemical groups with the tuning of reaction temperature and time.^52^ Previously, it was noted that nano-sized carbon nanodots (CNDs) accompany micro-sized carbon micro spheres (CMSs) as paired products. CMSs frequently originate from carbonized sediment, and CNDs are dispersed in the supernatant. Nitrogen–sulfur-doped CQDs show green fluorescence that shifts to strong blue fluorescence once diluted, a distinct phenomenon of concentration-dependent luminescence correlated to the particle size. Van der Waals forces cause nanoparticles to clump together at high concentrations and spread out when the concentration drops, resulting in a decrease in particle size and different emission patterns. Thus, particle size affects the conjugated π structure of CDs, which in turn affects optical properties and fluorescence emission, correlating well with our results.^53^ Bright blue and pacific blue fluorescence can be observed from SB and MW synthesized CQDs, respectively, under UVA but appear yellow and transparent, respectively, in white light, as shown in Fig. 4a and b. The UV-vis absorption spectra of both CQDs are consistent with previous studies, as depicted in Fig. 4c. The bands for the Glu–Trp solution exhibit a strong absorption peak at 219 nm, also shared by CQD-MW, while a hump in the region of 350–400 nm is overlapped by CQD-SB accompanied by a peak at 228 nm. A shift in the glucose peak from 219 nm to 228 nm can be observed in CQD-SB. Since glucose is a colorless molecule and its electronic transition lies in the far ultraviolet spectrum, a pronounced peak at ∼219 nm is attributed to glucose absorption, as previously reported for glucose ∼200.^54^ However, the indole ring of Trp is absorbed strongly in the near-ultraviolet wavelength of the spectrum, with an absorption maximum at 280 nm ^55^ representing the similar region spanning from 250 to 300 nm in our study.

Absorption bands in the short wavelength range of 214–300 nm have been described in previous studies, which take place in the carbonized cores of CQD-SB and CQD-MW initiated from the aromatic π system. These bands correspond to an aromatic π–π* unsaturated bond (C=C) transition of sp^2^ domains and the related electron transitions in the CDs that contain oxygen. The n–π* transition of the C=O bonds (associated with the sp^3^ carbon system) and C=N bonds is represented by the large absorption bands at 340 and 410 nm in the longer wavelengths present on the surface.^21,29,56–58^ The absorption bands up to 280 nm could be assigned to the π–π* transitions of the C=C and C=N bonds.

CIE 1931 color coordinate software was used to plot the fluorescence emission spectra from excitation at 360 and 450 nm from both CQD-SB and CQD-MW, respectively (Fig. 4d). CQD-SB was more towards the higher energy area compared to CQD-MW. Upon excitation at 450 nm, the difference in energy area between CQDs is negligible, and both fall in the same green region. This CIE pattern indicates that CQD's fluorescence behavior, including QCE (quantum confinement effect), QSE (quantum size effect), surface state and molecule state, are determined by the carbon core. With an increase in the reaction temperature, the carbon core is formed by the dehydration and carbonization of raw molecules, exhibiting unique photoluminescence (PL) and photostability.^52^ Moreover, the formation of secondary derivatives due to tryptophan doping and the size of the π-conjugate domain play a crucial role in PL behavior. The band-gap energy is influenced by the fragment size, and it decreases as the CD size increases. Furthermore, the surface state is determined by the connection between the chemical groups and the surface of the carbon backbone.^59^

**Fig. 5:**
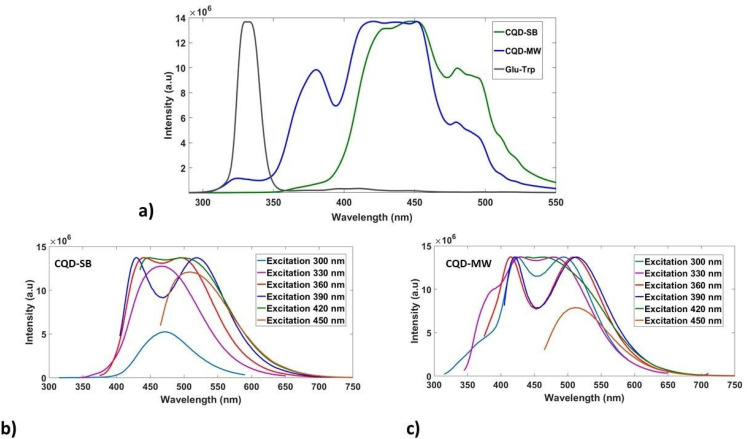
(a) Variation in synchronous fluorescence spectra of precursors (Glu–Trp), CQD-SB and CQD-MW produced through sand bath and microwave. (b) and (c) Photoluminescence emission spectra of CQD-SB and CQD-MW showing dual emission in the blue and green regions at different excitation wavelengths (300–450 nm) with an increment of 30 nm.

These unique concentration-dependent dual emissive CQDs were subjected to synchronous fluorescence scanning spectroscopy at 30 offset with 5 slit sizes optimized after a series of experimentation. Fig. 5a–c shows the synchronized fluorescence and emission spectra of CQDs (SB and MW), ranging from 400 to 460 nm in aqueous solution, which shows spectrum diversity compared with the Glu–Trp solution. The region around 400 nm in Glu–Trp showed very low intensity humps, while an intense peak was observed around 340 nm. Dual fluorescence emission peaks were displayed in the blue and green regions by both CQDs. It has been reported that functional groups, such as C=O (carbonyl) and C=C (conjugated sp^2^ domains), contribute significantly to fluorescence intensity and stability, causing emission in the blue and green regions.^60^ The emission maxima of the main emission bands (416–450 nm) in CQD-SB are red-shifted compared to CQD-MW, probably originating from the conjugated electron system of complicated MRPs owing to prolonged heating at high temperatures. The blue-green emission intensity ranging from 480 to 496 nm was also higher in CQD-SB.

Interestingly, CQD-MW exhibited tryptophan-like fluorescence emission even though there were no corresponding absorption features in the range of 250–300 nm, as shown in the absorption spectra (Fig. 4c). This suggests that functional fluorescence routes resembling Trp emission are still active maybe as a result of surface-bound fragments or dopant-induced surface states, even though the structural integrity of the indole chromophore may not be maintained. In contrast, CQD-SB showed that absorption bands aligned with Glu–Trp; however, the absence of its distinctive emission bands at 323 nm and 380 nm in synchronous fluorescence spectra (Fig. 5a) suggests a structural and electronic change during synthesis, possibly resulting from deeper carbonization caused by extended heating or the embedding of fluorophores that quench the native fluorescence. These observations highlight the effect of synthesis methods on the optical characteristics of Trp-integrated CQDs. Tryptophan exhibits emission ranging from 270 to 470 nm in solution with a maximum at 350 nm when excited at 280 nm, but the spectrum is influenced by the polarity of the solvent and blue-shifted if the tryptophan residue is buried in any hydrophobic environment.^61,62^ This shift in the spectrum may be attributed to the carbonyl amine condensation reaction between glucose and tryptophan, leading to the formation of a complex mixture of Maillard reaction products.^63^

The PL emission spectra of both CQDs are presented in Fig. 5b and c with an excitation range of 300–450 nm. The fluorescence emission behavior of the synthesized CQD-SB also reveals a distinct excitation-dependent mechanism. Upon excitation at shorter wavelengths (300–330 nm), a single emission band at 475 nm originates, suggesting emission from core-localized π–π transitions. In contrast, excitation at longer wavelengths (360–390 nm) induces dual emission bands at 420 nm and 530 nm, indicating the activation of surface-attached emissive traps. The appearance of dual emission (420 nm and 530 nm), showing simultaneous blue and red shift upon longer wavelength excitation, reflects a heterogeneous population of emissive sites within the CQDs, as shown in the PL spectra (Fig. 5b). However, CQD-MW likely has shallower surface traps, allowing for dual emission even at high excitation energies (300–330 nm), as depicted in Fig. 5c. Multiple emissive sites playing a role in dual emission might be energetically closer to the core states possibly owing to less passivation, faster carbonization, or incomplete nitrogen incorporation in CQD-MW.

**Fig. 6:**
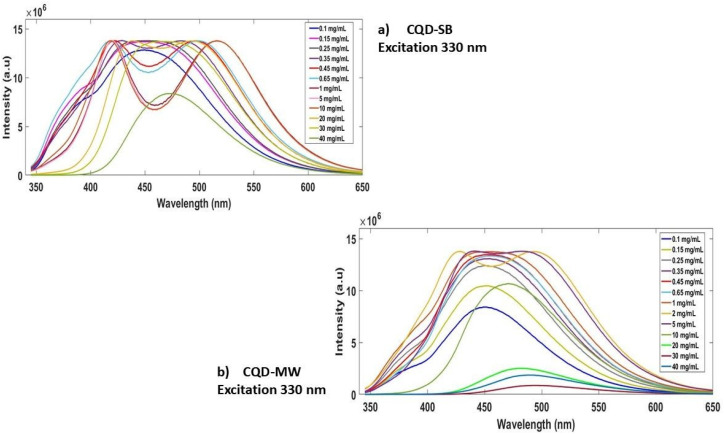
Photoluminescence emission spectra of CQDs at 330 nm. (a) CQD-SB at varying concentrations (0.1–40 mg mL^−1^). (b) CQD-MW at varying concentrations (0.1–40 mg mL^−1^); both CQDs indicate distinct emissive states, followed by spectral broadening and reduced intensity at higher concentrations.

The concentration-dependent emission at 330 nm, ranging from 0.1 to 40 mg mL^−1^ in both CQDs, showed a strong fluorescence emission response at certain concentrations (Fig. 6). Dual peaks in CQD-SB seem to originate from both the surface state and core emission, which appear at 0.35 mg mL^−1^ and persist until 10 mg mL^−1^, leading to distinct emissive states. The dual emission began to red shift and diminished at 20 mg mL^−1^ with a conversion to a low intensity single band at 40 mg mL^−1^ due to self-quenching and reabsorption that completely mask the dual emission at high concentration (Fig. 6a). However, in CQD-MW, the increasing intensity of a single band was observed to 0.65 mg mL^−1^ with a bifurcation of dual peaks at 1 mg mL^−1^, which is more pronounced at 2 mg mL^−1^. PL intensity dropped sharply >2 mg mL^−1^ with red shift and conversion to a single band owing to reabsorption and aggregation-induced quenching. These PL bands in the visible region (350–600 nm) are attributed to the cumulative effect of the π–π* transitions of the intrinsic carbon core, as well as the n–π* transitions between various surface groups on the surface of CQD-SB and CQD-MW.^64^ The dual emission in CQDs may be due to the independent multiple emission centers or the transitions of electrons confined in the carbon core.^65^ Another study on CDs synthesized solely from glucose using hydrothermal synthesis reported that tunable fluorescence from CDs is attributed to particle size variations and functional hydroxyl and carboxyl groups on the surface.^66^

In general, the chemical structure of the fluorophore decides the energy spacing between the ground and the excited energy levels; hence, heating generates a series of complex advanced glycation end products^67,68^ with a characteristic fluorescent emission in the range of 420–440 nm when excited at 340–370 nm.^69,70^ The absorbance values at 420 nm may be associated with the products in the final stage of MRPs, while the value at 294 nm is reflective of the intermediate MRPs. Moreover, the 294/420 nm absorbance ratio was suggestive of the extent of polymerization.^71^ It is important to note that the reactions that occur during the advanced stage of the Maillard reaction, especially the condensation reaction, are analogous to the hydrothermal synthesis process of CDs prepared from molecular precursors, such as citric acid and ammonia solutions. Some products from the Maillard reaction can emit fluorescence when irradiated with UV light, as previously reported.^72^ A high absorbance value and red shift were observed owing to the introduction of a conjugated π–electron system in Glu–Trp solution, which extended the conjugate electron system of MRPs and reduced the energy gap for the π–π* transitions, resulting in enhanced fluorescence intensity with a red-shift.^68^ The fluorescence emission of CQDs produced by microwave-aided synthesis is caused by fluorophores that are present on their surfaces and correlates with tryptophan doping.^73^

**Fig. 7:**
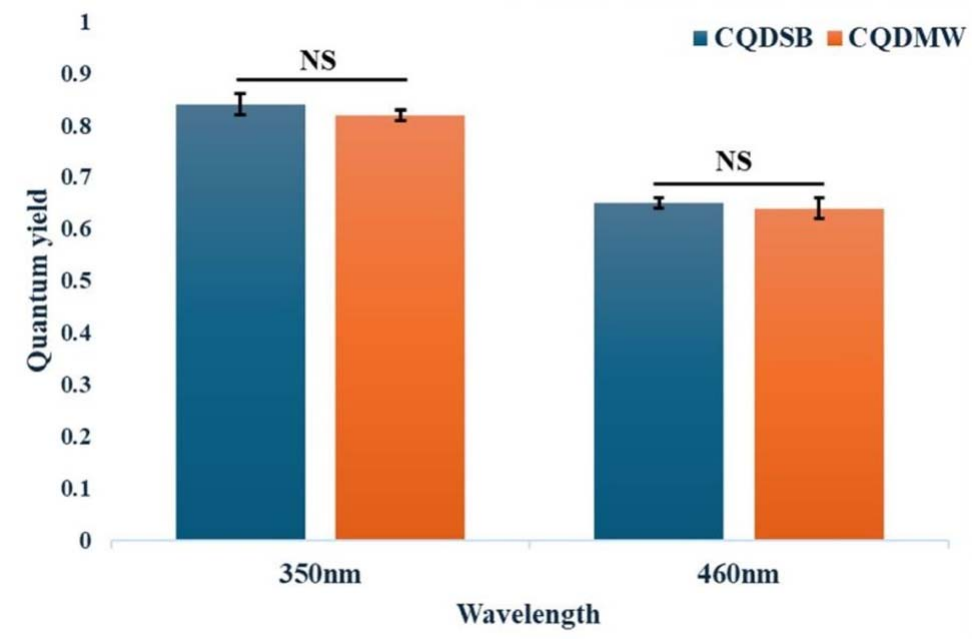
Quantum yield (QY) comparison of CQD-SB and CQD-MW at excitation wavelengths of 350 nm and 460 nm. Coumarin 450 (QY = 0.83) and fluorescein (QY = 0.97) were used as reference standards for 350 nm and 460 nm excitation, respectively. No statistically significant difference (NS) in QY was observed between the two CQD samples at either wavelength (*P* = 0.10 and *P* = 1.00, respectively). Error bars represent the standard deviation (SD) from three independent measurements (*n* = 3).

Fig. 7 depicts a comparative analysis of the quantum yield (QY) of CQD-SB and CQD-MW at two excitation wavelengths (350 nm and 460 nm). Coumarin 450 was employed as the standard reference dye at 350 nm excitation, and both CQD-SB and CQD-MW exhibit high fluorescence efficiencies, with QY exceeding 0.80. Similarly, at 460 nm, where Fluorescein was used as the standard, the QYs for both samples remain relatively high, ranging between 0.70 and 0.75. No statistically significant difference was observed in the QY between CQD-SB and CQD-MW at either excitation wavelength despite the use of different synthesis approaches. Although CQD-SB showed improved colloidal stability and greater fluorescence compared to CDQ-MW, its quantum yield remained similar. This might be due to the saturation of emissive states and persistent non-radiative decay channels that limit photon conversion efficiency across synthesis methods, as discussed previously.^74^ Though numerically close, the enhanced fluorescence of CQD-SB is likely influenced by other factors beyond quantum yield alone owing to the presence of additional oxygen-rich carbonyl groups (C=O) that promote radiative recombination and surface passivation as compared to CQD-MW (Fig. 2a and b), which amplified emission intensity without necessarily increasing conversion efficiency. A study reported that the QYs of CQDs can be enhanced using surface passivation, which eliminates emissive traps from the surface.^75^

#### Machine learning tool

**Fig. 8:**
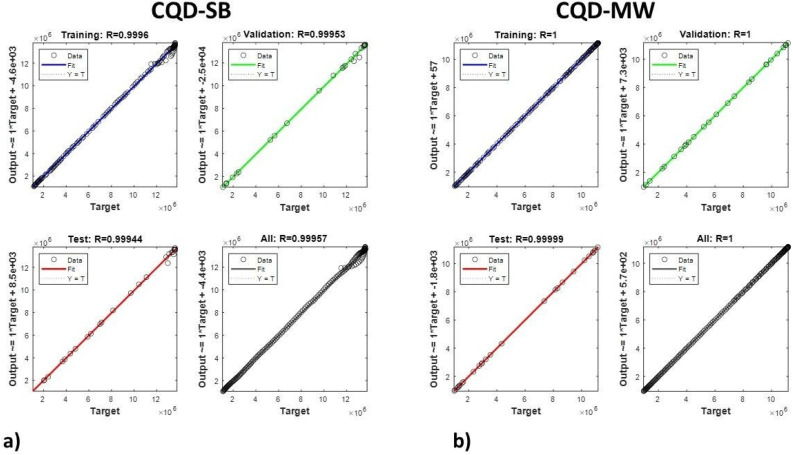
Regression plot of the Levenberg–Marquardt (LM) algorithm for fluorescence analysis of (a) CDQ-SB and (b) CQD-MW validated against two dyes: Coumarin 450 and Fluorescein.

The LM algorithm was implemented to fit the nonlinear disintegration of fluorescence intensity with high accuracy. Emission spectra of both CQDs were subjected to the LM algorithm along with Coumarin 450 and Fluorescein as references for dual emission. The data were split into three sets: 70% for training, 15% for validation, and 15% for testing along with the references. The model was fitted using the training data; its internal parameters were adjusted using the validation set, and test data provided an objective assessment of the model's performance on unidentified/unknown data. Regression plots (Fig. 8a and b) depict how the predicted values correspond with actual target values, with *R*^2^ values for coefficients reported at 0.9997 for training, 0.9999 for validation, 0.99998 for testing, and 0.9998 total. The high *R*^2^ values point toward high correlation and little prediction error, indicating robust model performance and generalization. The fitted lines in the regressions follow very closely to the ideal line *Y* = *T*, evidencing precise predictive activity in every data subset. These results are in line with the optical properties of CQD-SB and CQD-MW, supporting the effectiveness of the LM algorithm in terms of fluorescence-based modeling. The studies highlighted its ability to model nonlinear photophysical responses using CQD-SB and CQD-MW with high accuracy, validating its use in this study as well.

#### Single cell imaging

**Fig. 9:**
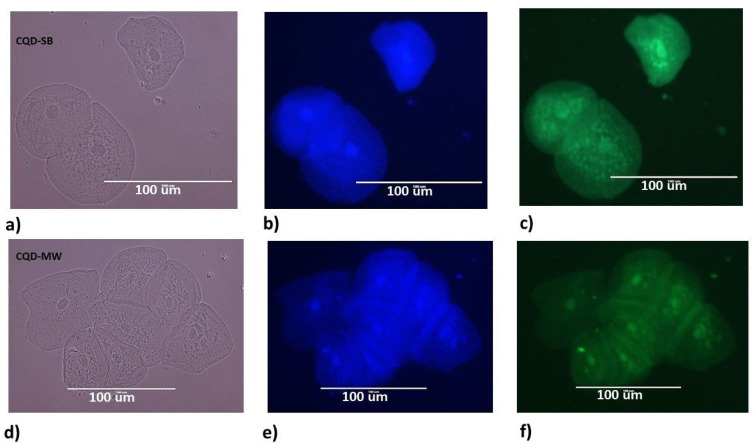
Fluorescence microscopy imaging of buccal mucosal cells using both CQDs: (a) and (d) under white light mode for CQD-SB and CDQ-MW; (b) and (e) under DAPI mode with excitation 357 nm, showing bright blue fluorescence; (c) and (f) under GFP mode excitation 395 nm, showing bright green fluorescence in CQD-SB compared to CQD-MW.

To investigate the applicability of CQDs as fluorescent biomarkers in a practical biological environment, excitation-dependent dual emissive CQD-SB and CQD-MW were used for imaging single cells from humans and plants. Buccal mucosal samples were easily obtained from the buccal cavity and incubated for 1 h with aqueous solutions of CQD-SB and CQD-MW, followed by fluorescence microscopy (Fig. 9a–f). When these samples were excited at 357 nm (DAPI mode), blue fluorescence was emitted, and upon excitation at 395 nm (GFP mode), the samples emitted green fluorescence. The buccal cells treated with CQD-SB showed brighter fluorescence localized within the cytoplasm and cell nucleus at both excitation wavelengths compared to CQD-MW. The results are consistent with the earlier findings that CDs might stain the cytoplasm, nucleus and cell membrane because of the charged CD surface that induced cell membrane penetration, which facilitates the CD-nucleus binding *via* electrostatic attraction.^33,76^

**Fig. 10:**
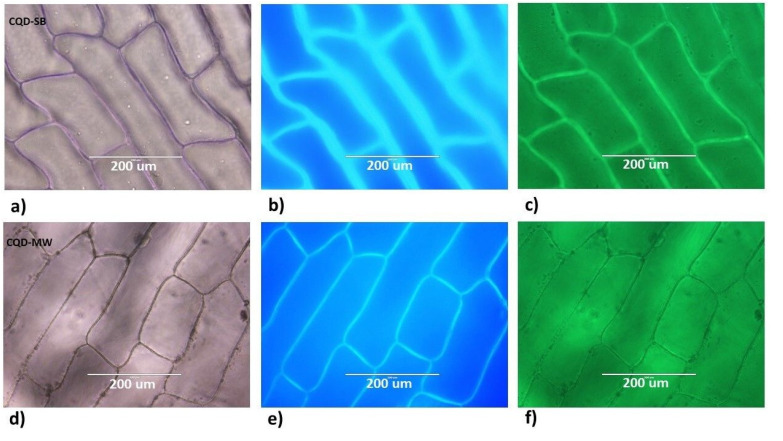
Fluorescence microscopy imaging of onion cells using both CQDs: (a) and (d) under white light mode for CQD-SB and CDQ-MW; (b) and (e) under DAPI mode with excitation at 357 nm, showing bright blue and intense fluorescence in CQD-SB compared to CQD-MW; and (c) and (f) under GFP mode excitation at 395 nm, showing bright green fluorescence in CQD-SB only.

An interesting observation was made when both CQDs were used to stain the onion epidermis cell layer, forming the outermost layer of cells in an onion bulb composed of pectin and cellulose. CQD-SB successfully stained the onion epidermal cell wall even obvious in white light but lacking in CQD-MW (Fig. 10a and d). Similarly, upon excitation at 357 nm, the localized fluorescence from these CQDs stained cells is observed; however, CQD-SB showed markedly stronger intensity compared to the CQD-MW (Fig. 10b and e). Upon excitation at 395 nm, enhanced green fluorescence was observed in CQD-SB-treated samples in contrast to CQD-MW with much fainted fluorescence (Fig. 10c and f). These results suggest that CQD-SB not only adheres to the cell wall but also permeates internal organelles and cytoplasmic region,^61^ hence presenting a strong potential to be used as a fluorescent probe for plant cell imaging.

#### Stability studies

**Fig. 11:**
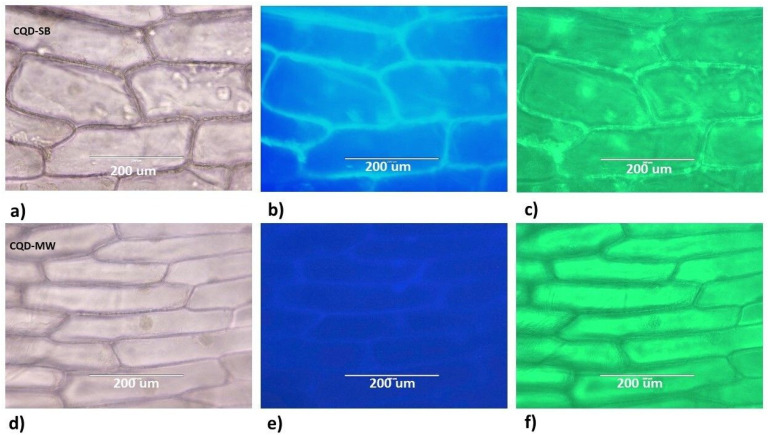
Fluorescence microscopy imaging of onion cells using 60 day-old CQDs; (a) and (d) under white light mode for CQD-SB and CDQ-MW; (b) and (e) under DAPI mode with excitation at 357 nm, showing bright blue and intense in CQD-SB but very faint in CQD-MW; and (c) and (f) under GFP mode excitation at 395 nm, showing diminished green fluorescence in CQD-SB but intense fluorescence in the cytoplasm of CQD-MW.

CQD-SB and CQD-MW stored at 4 °C were evaluated after 60 days for post storage fluorescence, photoluminescence and staining efficiency. Given that the long-term stability of CQDs is essential for their use in practical applications, onion epidermal cells were used owing to the structural rigidity of the cell membrane due to the presence of pectin and cellulose. An additional hour of staining was required after 60 days for adequate staining, as the fluorescence intensity of the CQDs dropped over time. CQD-SB retained its consistent binding pattern when excited at both 357 and 395 nm wavelengths (Fig. 11b and c), while CQD-MW showed diminished blue fluorescence, as shown in Fig. 11e. Interestingly, upon excitation at 395 nm, onion epidermal cells stained with CQD-MW did not localize in the cell wall, but internal cells containing the cytoplasm fluoresced green, hence suggesting its potential as a selective cytoplasmic fluorescent probe. Prolonged storage might cause particle aggregation, altering their size or rearrangement of functional group affecting diffusion properties could reduced affinity for cell walls.

**Fig. 12:**
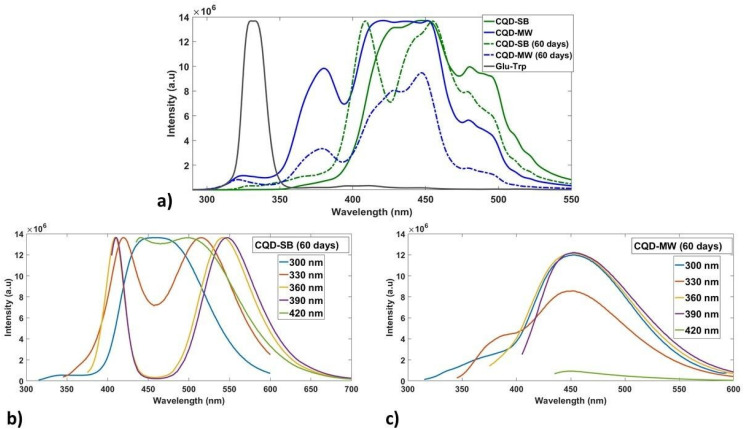
Synchronous fluorescence spectra and emission spectra of 60 day-old CQDs. (a) Variation in synchronous fluorescence spectra of precursors (Glu–Trp), CQD-SB and CQD-MW in solid lines and 60 day-old in dashed line, showing low intensity in CQD-MW. (b) and (c) Photoluminescence emission spectra of CQDs showing dual emission in the blue and green regions by CQD-SB but diminished fluorescence intensity in the blue region of CQD-MW at different excitation wavelengths with an increment of 30 nm.

Synchronous fluorescence spectra of stored CQDs revealed reduced intensity in CQD-MW (Fig. 12a), while CQD-SB spectra slightly shifted towards shorter wavelengths and split into a 400 nm pronounced peak and a broad band ranging from 430 to 510 nm. PL emission spectra of stored CQD-SB showed well-defined dual emission peaks from 400 to 450 nm and 500 to 650 nm when excited at different wavelengths from 300 to 420 nm (Fig. 12b). In contrast, CQD-MW showed disappearance of dual peak behavior but changed into a low intensity broad band extending from 350 to 550 nm (Fig. 12c) probably due to particle aggregation over time. Aging in CQD-SB may enhance emission peaks, as shown in Fig. 12a, which might happen due to structural rearrangements strengthening the coupling between emissive sites. However, cell staining with CQDs required extended incubation time after 60 days, and the fluorescence intensity was notably lower than that of freshly prepared samples probably due to surface-state degradation, leading to weaker fluorescence in staining despite improved internal energy transfer.

Microwave heating offers advantages, such as accelerated heating, ease of use, better homogeneity in temperature, and efficient dielectric coupling transforming electromagnetic radiation into heat without exceeding a temperature above 100 °C.^77^ Despite the advantages of microwave-assisted synthesis, CQD-MW showed reduced structural and fluorescence stability. However, sand bath-assisted synthesis maintained moisture owing to the reflux mechanism and uniform heat that produced more stable CQDs with complete carbonization of the graphitic core enriched with carboxyl and amine groups.

## Conclusion

These findings show that, although both samples were produced from the same precursor, the thermal conditions, *i.e.* sand bath and microwave heating and the associated energy input significantly affect the nucleation, growth, and surface features of the synthesized CQDs. The variation in morphological properties highlights the importance of tryptophan doping and the synthesis approach in modifying the physicochemical and optical behavior of CQDs. The photoluminescence of these CQDs is excitation and concentration dependent, but CQD-SB was found to be more photostable compared to microwave-assisted CQDs. The enhanced optical properties of CQD-SB are due to the formation of dual emissive carbonized products, as validated with fluorescence spectroscopy and bioimaging. Moreover, it is evident that the size effect could be the primary cause of the variations in the fluorescence characteristics of CQDs at various concentrations. By combining these fluorescent materials with other doping agents and changing the temperature and time duration, new optical properties can be introduced or enhanced for extensive applications in the future.

## Ethical statement

All experiments were performed in accordance with the Guidelines of “EU No. 536/2014” and were approved by the ethics committee at “NILOP (PIEAS)”. Samples were self-donated, and human participants were not involved.

## Conflicts of interest

There are no conflicts to declare.

## Data Availability

The data underlying this study are available in the published article, moreover the computational data supporting the findings of this study, including input files will be made available from the corresponding author upon reasonable request.
